# Cancer and COVID-19: unravelling the immunological interplay with a review of promising therapies against severe SARS-CoV-2 for cancer patients

**DOI:** 10.1186/s13045-023-01432-6

**Published:** 2023-04-13

**Authors:** Yan Leyfman, Nancy Emmanuel, Gayathri P. Menon, Muskan Joshi, William B. Wilkerson, Jared Cappelli, Timothy K. Erick, Chandler H. Park, Pushpa Sharma

**Affiliations:** 1 Icahn School of Medicine at Mount Sinai South Nassau, Rockville Centre, NY USA; 2 Hospital das Clínicas of the Faculty of Medicine of the University of São Paulo, São Paulo, Brazil; 3 Tbilisi State Medical University, Tbilisi, Georgia; 4 Dickinson College, Carlisle, PA USA; 5 UTHSC Nashville, Nashville, TN USA; 6 Dana-Farber Cancer Institute, Boston, MA USA; 7 Norton Cancer Institute, Louisville, KY USA; 8 Department of Anesthesiology, Uniformed Services University of the Health Sciences, 4301 Jones Bridge Road, Bethesda, MD 20814 USA

**Keywords:** COVID-19, SARS-CoV-2, Cancer, Hypoxia, IL-6, Therapeutics

## Abstract

Cancer patients, due to their immunocompromised status, are at an increased risk for severe SARS-CoV-2 infection. Since severe SARS-CoV-2 infection causes multiple organ damage through IL-6-mediated inflammation while stimulating hypoxia, and malignancy promotes hypoxia-induced cellular metabolic alterations leading to cell death, we propose a mechanistic interplay between both conditions that results in an upregulation of IL-6 secretion resulting in enhanced cytokine production and systemic injury. Hypoxia mediated by both conditions results in cell necrosis, dysregulation of oxidative phosphorylation, and mitochondrial dysfunction. This produces free radicals and cytokines that result in systemic inflammatory injury. Hypoxia also catalyzes the breakdown of COX-1 and 2 resulting in bronchoconstriction and pulmonary edema, which further exacerbates tissue hypoxia. Given this disease model, therapeutic options are currently being studied against severe SARS-COV-2. In this study, we review several promising therapies against severe disease supported by clinical trial evidence—including Allocetra, monoclonal antibodies (Tixagevimab–Cilgavimab), peginterferon lambda, Baricitinib, Remdesivir, Sarilumab, Tocilizumab, Anakinra, Bevacizumab, exosomes, and mesenchymal stem cells. Due to the virus’s rapid adaptive evolution and diverse symptomatic manifestation, the use of combination therapies offers a promising approach to decrease systemic injury. By investing in such targeted interventions, cases of severe SARS-CoV-2 should decrease along with its associated long-term sequelae and thereby allow cancer patients to resume their treatments.

## Introduction

In December 2019, a respiratory illness surged in Wuhan, China, and by the first week of January, the investigations attributed these cases to an infection caused by a coronavirus. This virus was initially named 2019 novel coronavirus (2019-nCoV), but the name was changed to severe acute respiratory distress syndrome coronavirus 2 (SARS-CoV-2) due to its similarities with the previously described severe acute respiratory distress syndrome coronavirus (SARS-CoV). The disease resulting from this novel coronavirus infection was named coronavirus disease 2019 (COVID-19) [1]. COVID-19 was declared a pandemic by the WHO in March 2020, and since then, more than 148 million cases have been recorded with more than 3.1 million deaths worldwide [2]. While the virus is mainly of respiratory origin, it can also act systemically resulting in multi-organ injury [3].

Since the start of the pandemic, efforts have been focused to better understand the pathogenesis SARS-CoV-2 with the aim of discovering targeted therapies. SARS-CoV-2 uses its spike protein to bind to the angiotensin converting enzyme-2 (ACE-2) receptors for entry into human cells. Because the ACE-2 receptor is expressed by many cell types, the virus can infect many organ systems (e.g., respiratory, cardiovascular, gastrointestinal, renal, and hepatobiliary) [4]. Other proteins have also been found to be involved in viral entry, including the CD147-spike protein, furin, and GRP78 receptors [5].

After invasion via the ACE-2 receptor, the host immune response leads to hypersecretion of interleukin-6 (IL-6) and various other inflammatory cytokines. This is termed cytokine release syndrome (CRS) and in severe cases, may lead to subsequent multi-organ failure [6]. Early work in cytokine research has demonstrated a link between various cytokine levels in the plasma and COVID-19 severity, suggesting the possibility of future prognostic stratification [7]. Additionally, viral load may also have implications in characterizing disease severity in hospitalized COVID-19 patients [8]. Monitoring the levels of IL-6 and TNFα has been proposed as a strategy to guide the management of COVID-19 patients as these levels were found to be significant predictors of severity and mortality [9]. More recent studies have validated the role of IL-6 levels as a prognostic indicator and have found that consistently increasing levels of CRP, WBC, TnI, and IL-6 are meaningful predictors of patient mortality [10].

Cancer patients are a particularly vulnerable population that require specialized care and treatment, including surgeries, chemotherapy, and radiation therapy, which may not have been readily available during the pandemic due to the need to prioritize COVID-19 patients and reduce the risk of virus transmission [11]. Many hospitals and clinics had to reduce or cancel non-urgent medical procedures to open up resources for COVID-19 patients, leaving many cancer patients without access to necessary medical care. Additionally, fear of exposure to the virus and lockdown measures may have caused cancer patients to avoid seeking medical attention, leading to delays in diagnosis and treatment. The unavailability of medical assistance for cancer patients during the pandemic can have serious consequences, including the progression of the disease, increased pain and suffering, and reduced chances of survival.

Severe SARS-CoV-2 places cancer patients at a disproportionately elevated risk of morbidity and mortality compared to patients without malignancy [7, 12]. Many confounding factors contributed to this observation in the cancer population including older age, smoking history, comorbid conditions, and antineoplastic therapies [13, 14]. Yet, a study comparing COVID-19-positive cancer and non-cancer patients showed that cancer is independently and strongly associated with a poor outcome in COVID-19 patients, as well as with increased hospitalization and a higher 30-day mortality [15, 16].

The aim of this study is to discuss the immunological interplay between SARS-CoV-2 and cancer, and how these two superimposed multisystem diseases can exacerbate each other, while also providing a unique avenue for novel therapeutic interventions. We discuss the mechanism of SARS-CoV-2 infection, associated CRS, mechanism of hypoxia, and their association with concomitant malignancy. Finally, we provide a discussion of multiple novel therapeutic modalities, including mesenchymal stem cell (MSC) therapy, exosomal therapy, monoclonal antibodies, and other targeted agents that have demonstrated promising efficacy in clinical trials.

## Mechanism for SARS-CoV-2 infection

Upon entering the body, SARS-CoV-2 has been understood to act via multiple pathways, both directly and indirectly to cause systemic inflammatory damage [17].

### Direct pathway for SARS-CoV-2 infection

Once SARS-CoV-2 enters the respiratory tract, the viral spike (S) cholesterol-laden glycoprotein binds to ACE2 on the surface of epithelial cells [18]. Proteolytic cleavage of the S protein by Type II transmembrane serine proteases 2 (TMPRSS2), furin, and cathepsin L promotes viral endocytosis. Although TMPRSS2 and ACE2 are present within the epithelial cells of most major organs, including the vasculature, kidneys, pancreas, gastrointestinal tract, eyes, and hepatocytes, a higher expression is present within the nasopharyngeal tract and lungs resulting in higher affinity for these tissues [5, 19, 20]. Viral invasion of non-epithelial cells, like the vascular smooth muscle cells of the heart, has also been observed [21]. Upon entry into host cells, SARS-CoV-2 co-opts host cellular machinery to replicate its genome and produce new virions. In the lungs, SARS-CoV-2 also stimulates the release of IL-6, a key cytokine involved in immune signal amplification, largely seen in those with severe disease [22–24].

**Fig. 1:**
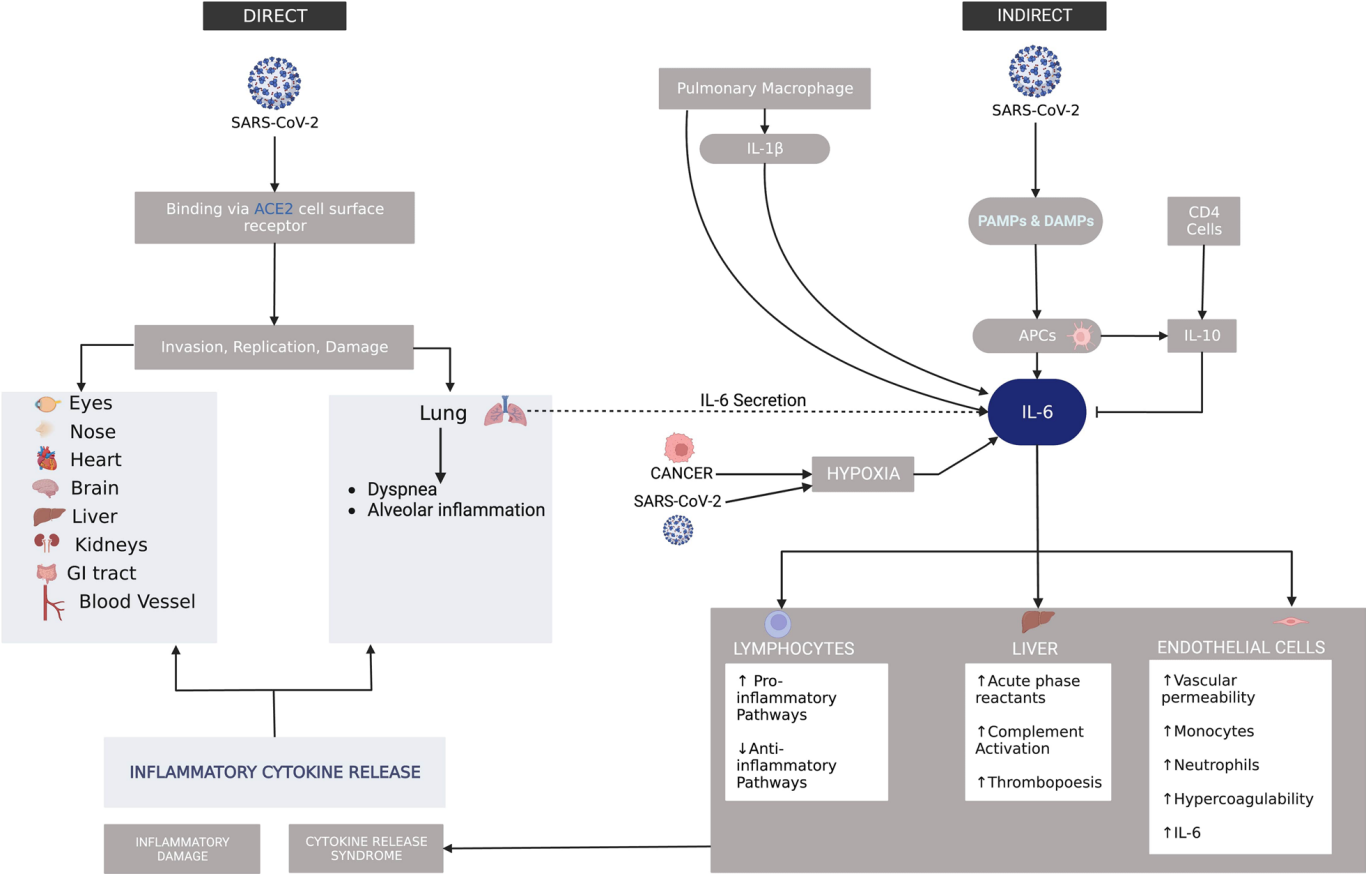
COVID-19 Cancer Model. SARS-CoV-2 acts via both a direct and indirect pathway to induce systemic injury. The virus enters the body via ACE2 receptors on the cell surface of most of the organs resulting in invasion, replication, and damage. It causes a hypercoagulable state within the blood vessels and dyspnea within the lungs, which also secrete IL-6 that goes into the indirect pathway. In the indirect pathway, SARS-CoV-2 acts via PAMPs and DAMPs to activate antigen presenting cells (APCs) to secrete IL-6. Additionally, the virus activates pulmonary macrophages which secrete IL-6 directly and/or via IL-1. Malignancy and severe SARS-CoV-2 induce hypoxia, which is a trigger for IL-6 secretion. IL-6 activates downstream pathways to promote a pro-inflammatory state. Ultimately, this leads to increased cytokine release resulting in systemic inflammatory damage. The image of SARS-CoV-2 was derived from the Centers for Disease Control and Prevention (CDC) website: https://phil.cdc.gov/Details.aspx?pid=23312

Although COVID-19 clinical presentations are diverse, the most common symptoms involve the respiratory tract presenting with anosmia and ageusia [25], which then can progress to dyspnea [26] and possibly respiratory failure. The resulting direct damage has broad clinical manifestations depending on the organ affected and can present with vascular hypercoagulability [27], myocardial infarction [28], renal failure [29], diverse neurological manifestations [30], and/or non-specific gastrointestinal symptoms [31] (Fig. 1).

### Indirect pathway for SARS-CoV-2 infection resulting in systemic injury

In addition to the direct pathway, SARS-CoV-2 also acts in an indirect manner resulting in systemic injury. Pyroptosis of damaged cells induces the release of damage-associated molecular patterns (DAMPs) and pathogen-associated molecular patterns (PAMPs) resulting in the activation of antigen presenting cells (APCs) consisting of macrophages, dendritic cells, and monocytes [32–34]. Activation of APCs results in increased production and secretion of IL-6 [32]. Additionally, pulmonary macrophages, the primary APC of the lungs, are mobilized and secrete IL-1β, which drive the activation of pro-inflammatory pathways and further upregulation of cytokines, including IL-6 [35]. Both SARS-CoV-2 and malignancy can induce hypoxia, which acts as a trigger for further IL-6 secretion [36, 37]. Although elevated IL-6 also upregulates the production and secretion of the anti-inflammatory cytokine IL-10 to regulate IL-6 expression, in the case of SARS-CoV-2, the production of pro-inflammatory IL-6 is overwhelmingly greater than that of IL-10, resulting in a net pro-inflammatory state [17] (Fig. 1).

IL-6 is a cytokine with pleiotropic activities with a cardinal role in downstream signal amplification. It results in an upregulation of pro-inflammatory pathways, downregulation of anti-inflammatory pathways, thrombopoiesis and hypercoagulability, production of acute phase reactants from the liver, vascular permeability, recruitment of monocytes and neutrophils, and complement activation [38, 39]. It also plays an important role in the adaptive immune response by stimulating antibody production and effector T cell development. This inflammatory cytokine release results in a cytokine release syndrome (CRS) that feeds back into the direct pathway resulting in systemic inflammatory injury.

### Interplay between malignancy, hypoxia & SARS-CoV-2

**Fig. 2:**
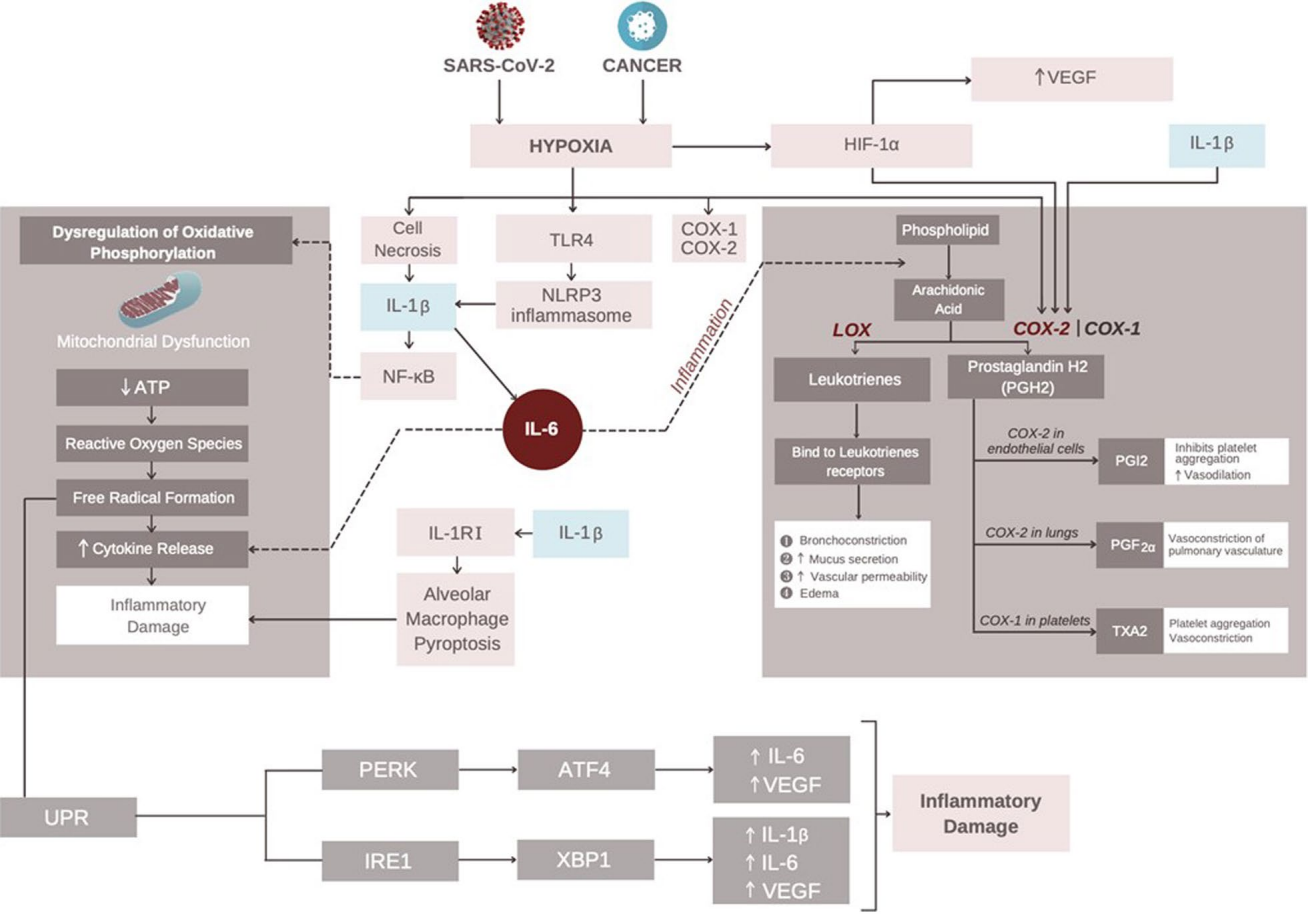
SARS-CoV-2 & Cancer-induced Hypoxia. SARS-CoV-2 and cancer induce hypoxia resulting in cellular necrosis and upregulation of the TLR4 pathway and HIF-1α. This results in increased secretion of IL-1β that stimulates NF-κB to cause mitochondrial dysfunction, secretion of reactive oxygen species and subsequent inflammation. IL-1β stimulates IL-1RI to cause alveolar macrophages pyroptosis to cause inflammatory damage. Hypoxia, IL-1β, and HIF-1α stimulate cyclooxygenase-2 (COX-2) which catalyzes the breakdown of arachidic acid into prostaglandin H_2_ (PGH_2_)_._ Further breakdown with COX-1 and 2 results in the production of prostacyclin, prostaglandin, and thromboxane. Hypoxic metabolic derangements result in the accumulation of misfolded proteins within the lumen of the endoplasmic reticulum (ER) resulting in activation of the unfolded protein response (UPR). This activates inositol-requiring enzyme 1 (IRE-1), which catalyzes the transcription of X-box binding protein 1 (XBP-1) and results in secretion of IL-1β and IL-6, which turn on downstream pro-inflammatory pathways. Additionally, hypoxia activates protein kinase R (PKR)-like endoplasmic reticulum kinase (PERK kinase) within the ER that stimulates activating transcription factor 4 (ATF4)-dependent transcriptional activation that results in secretion of IL-6 to promote inflammation. Additionally, HIF-1α, ATF4, and XBP-1 stimulate the release of vascular endothelial growth factor (VEGF). VEGF contributes to pulmonary inflammation and promotes vascular permeability resulting in pulmonary edema, which further exacerbates tissue hypoxia. The image of SARS-CoV-2 was derived from the Centers for Disease Control and Prevention (CDC) website: https://phil.cdc.gov/Details.aspx?pid=23312

COVID-19 with low oxygen saturations portends a poor prognosis [40]. Some studies have termed this “silent hypoxemia,” yet no mechanisms to explain this have been devised. Similarly in cancer, hypoxia alters cancer cell metabolism and contributes to therapy resistance by inducing cell quiescence [41]. This occurs when there is an imbalance in oxygen demand resulting in cell necrosis with activates the TLR4 pathway and increases the production of IL-1β and hypoxia-inducible factor one alpha (HIF-1α) (Fig. 2) [42–44]. Lipopolysaccharide (LPS) acts through TLR4 to activate the Nlrp3 inflammasome, which results in increased secretion of IL-1β. IL-1β through the IL-1 receptor I (IL-1RI) promotes alveolar macrophage pyroptosis resulting in inflammatory damage [45]. IL-1β also stimulates IL-6 which promotes the downstream activation of nuclear factor κB (NF-κB) [46, 47]. NF-κB causes dysregulation of oxidative phosphorylation which leads to the production of reactive oxygen species (ROS) that contributes to cytokine release and inflammation [48, 49]. Clinical studies are presently ongoing to develop SARS-CoV-2 prognostication assays and associations with post-acute sequelae of COVID-19 (PASC) using the information from this pathway [50–52]. A recent study found that elevated IL-1β, IL-6, and TNF were associated with PASC. Additionally, clinical trials are ongoing to find novel drug combinations to better control severe SARS-CoV-2 [53, 54].

IL-1β also acts via the phosphatidylinositol 3-kinase/AKT pathway to stimulate IL-6 secretion [55]. This promotes systemic inflammation through activation of pro-inflammatory pathways and stimulates the conversion of phospholipids into arachidonic acid [56]. Hypoxic conditions induce expression of cyclooxygenase-1 & 2 (COX-1 and 2) [57, 58]. Although the role of HIF-1α in the regulation of inflammation is debatable [59–61] under hypoxic conditions, it upregulates production of cyclooxygenase-2 (COX-2), just like IL-1β [62]. COX-1 and 2 then catalyze the conversion of arachidonic acid into prostaglandin H_2_ (PGH_2_). In platelets, COX-1 promotes further conversion into thromboxane A2 (TXA_2_) which increases platelet aggregation and vasoconstriction. However, in the endothelium, COX-2 stimulates the conversion into prostaglandin I_2_ (PGI_2_), which acts to inhibit platelet aggregation and promotes vasodilation [63]. Within the pulmonary vasculature, hypoxia past day 14 resulted in pulmonary hypertension and thrombosis due to increased production of PGI_2_ and TXA_2_ [64] and chronically to increase production of PGF_2α_ (largely responsible for vasoconstriction) [65]. Thus, hypoxia can also promote a hypercoagulable state.

Hypoxia’s metabolic derangements result in the accumulation of misfolded proteins within the lumen of the endoplasmic reticulum (ER). In response, cells activate a transcriptional program, known as unfolded protein response (UPR), that stimulates the activation of inositol-requiring enzyme 1 (IRE-1), which catalyzes the splicing and transcription of X-box binding protein 1 (XBP-1). XBP-1 stimulates release of IL-1β and IL-6, which turn on downstream signaling pathways that result in inflammatory damage [66]. Additionally, hypoxia also activates protein kinase R (PKR)-like endoplasmic reticulum kinase (PERK kinase) within the ER that stimulates activating transcription factor 4 (ATF4)-dependent transcriptional activation that results in downstream secretion of IL-6 that contributes to further inflammation [67].

ARDS and dyspnea result in pulmonary hypoxia, which stimulates the release of HIF-1α that upregulates vascular endothelial growth factor (VEGF) expression. VEGF expression is also stimulated by transcriptional activation of ATF4 and XBP-1. VEGF is a signal transduction factor that can trigger downstream immunomodulatory functionality after binding to the VEGF receptor resulting in balancing angiogenesis and immunosuppression [68]. In SARS-CoV-2 infected tissues, VEGF contributes to pulmonary inflammation and promotes vascular permeability resulting in pulmonary edema, which further exacerbates tissue hypoxia [69].

Studies have shown that higher cytokine levels of IL-1β, IL-6, TNF, IL-8, and IL-17 are associated with advanced stages of malignancy [70, 71]. Their upregulation in severe SARS-CoV-2 could help to explain the worsening outcomes for some cancer patients and could be utilized as targets for therapeutic intervention.

Although IL-6 is the primary cytokine in the interplay between SARS-CoV-2 and cancer, there are others involved as well currently under investigation. IFN-1, a prominent inhibitor of tumorigenesis and promoter of apoptosis, has been observed to be upregulated in patients with severe infection with TNF and IL-1 to drive inflammation and contribute to worsening infection [72]. Neutrophil extracellular traps (NETs), associated with the initiation of mesenchymal transition state and potentiation of migratory and invasive properties of cancer cells, have been shown to promote the production of pro-inflammatory cytokines in SARS-CoV-2 pulmonary disease leading diffuse alveolar damage and CRS. Severe SARS-CoV-2 has been associated with NET dysfunction leading to the development of immunothrombosis [73]. Studies from patients with severe SARS-CoV-2 have shown that carcinoembryonic antigen cell adhesion molecule (CEACAM1), a critical immune checkpoint receptor, is overexpressed and has been associated with HAVCR2, an immune checkpoint marker. This suggests that CEACAM1 might provide increased susceptibility for SARS-CoV-2 in cancer patients due to its interaction with HAVCR2 [74].

### Hypercoagulability

**Fig. 3:**
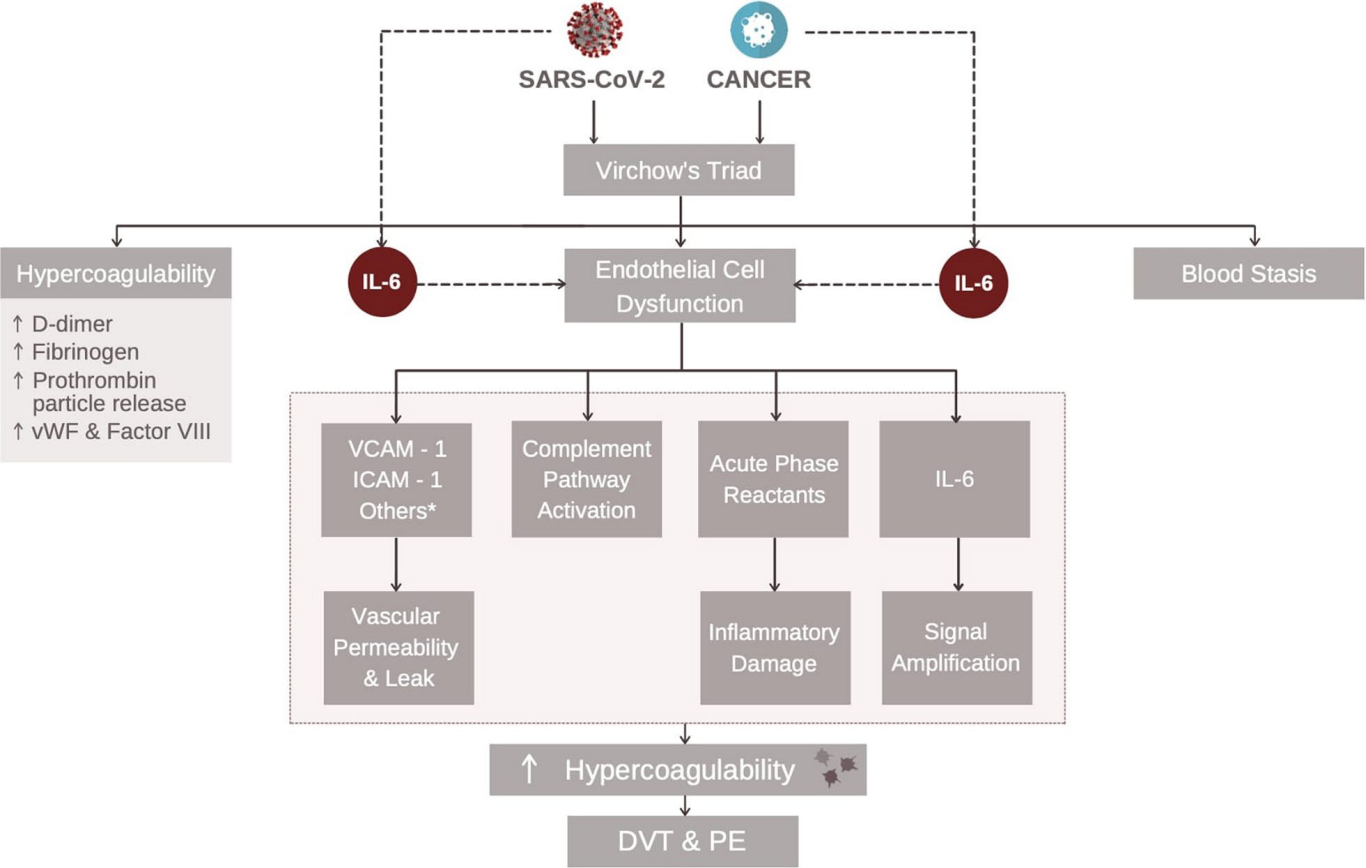
Hypercoagulability in Cancer & SARS-CoV-2. SARS-CoV-2 and cancer stimulate IL-6 secretion which causes endothelial cell dysfunction. This results in increased vascular permeability, complement activation, and inflammatory damage due to secretion of Acute Phase Reactants. Both SARS-CoV-2 and cancer contribute to Virchow’s Triad that, in addition to endothelial cell dysfunction, also includes blood stasis due to prolonged hospitalizations and hypercoagulability due to release of pro-coagulable factors. Ultimately, this results in a hypercoagulable state increasing the risk for DVTs and PEs. The image of SARS-CoV-2 was derived from the Centers for Disease Control and Prevention (CDC) website: https://phil.cdc.gov/Details.aspx?pid=23312

Hypercoagulability manifestations of pulmonary emboli and deep vein thromboses have been observed in 30% of COVID-19 patients along with the presence of prothrombotic autoantibodies in patients with severe SARS-CoV-2 [75, 76]. A study in cancer patients with severe SARS-CoV-2 showed that hospitalized patients with malignancy had a 34% thromboembolic risk with an incidence of 9.3% largely due to these patients already taking an anticoagulant [77]. Incidence was increased to 11% if these patients were admitted into the ICU. Multiple clinical studies have demonstrated greater platelet activation, reactivity, and aggregation in COVID-19 patients compared to healthy blood donors and patients with non-COVID-19 ARDS [78]. Mechanistically, this increased thromboembolic risk can be explained by an upregulation of IL-6 production, especially in those with malignancy and/or severe forms of SARS-CoV-2 resulting in increased trans-signaling in endothelial cells (Fig. 1) that increases permeability resulting in edema, as well as showing signs of vasculitis [79] in post-COVID-19 patients which could lead to the development of thrombosis [5]. IL-6 also acts on the liver to promote TPO secretion to stimulate thrombopoiesis and contributes to endothelial cell dysfunction that stimulates the release of vasodilatory molecules, including ICAM-1 & VCAM-1 [80, 81]. This results in vascular permeability (Fig. 3). Endothelial injury also results in the activation of the alternative and lectin complement pathways and release of acute phase reactants that result in inflammatory damage [82, 83].

Although the risk factors for cancer-associated thrombosis are multifactorial, studies have shown that cancer patients have higher circulating levels of coagulation factors and tissue factor. Tissue factor induces a pro-coagulable environment, directly activates Factor X of the coagulation cascade, and promotes angiogenesis with release of prothrombotic factors, including d-dimer, fibrinogen, Factor VIII, vWF, prothrombotic microparticles, and anionic phospholipids to foster a hypercoagulable state [84, 85]. Both malignancy and SARS-CoV-2 increase the risk of hospitalization resulting in an increased risk of blood stasis. Additionally, both conditions increase the risk of endothelial cell damage and dysfunction. The presence of hypercoagulability, stasis, and vascular endothelial damage are risk factors for Virchow’s triad, which contributes to a pro-coagulable state that increases the risk for pulmonary emboli (PE) and deep vein thromboses (DVTs) (Fig. 3).

## Therapeutic intervention

Although vaccines have demonstrated promising preventative efficacy against SARS-CoV-2, therapies are needed once a patient becomes symptomatic with COVID-19. Studies utilizing single-agent therapies have demonstrated limited efficacy, especially in patients with severe disease [86]. This is likely attributed to the inhibition of SARS-CoV-2 solely through the direct pathway—resulting in uninhibited systemic inflammation with subsequent inflammatory damage by the indirect pathway. Since SARS-CoV-2 acts on both pathways, it is important to consider the use of combination therapies that can simultaneously inhibit both viral replication and systemic inflammation at multiple points simultaneously.

### Convalescent plasma

Convalescent plasma provides passive immunity via neutralizing antibodies against the infectious pathogen. Initially, convalescent plasma was thought to play a role in immunocompromised patients who are unable to produce an adequate number of antibodies. However, several of the trials were abandoned because of futility or poor recruitment. One trial was stopped early after emergency use authorization was granted for convalescent plasma in the USA. In a study of 1225 participants (NCT04373460), most of whom were unvaccinated, the use of convalescent plasma within 9 days after symptomatic onset reduced the risk of disease progression leading to hospitalization [87]. However, in a study of 228 patients with severe SARS-CoV-2 (NCT04383535; NCT04884477), convalescent plasma did not provide an improvement in clinical status or overall mortality [88, 89]. However, a recent meta-analysis of three randomized clinical trials enrolling 1487 participants and five controlled studies looking at immunocompromised patients with COVID-19 suggested that COVID-19 convalescent plasma was associated with a mortality benefit [90]. This is in line with the US FDA’s present recommendations for the immunocompromised with COVID-19.

### Allocetra

**Fig. 4:**
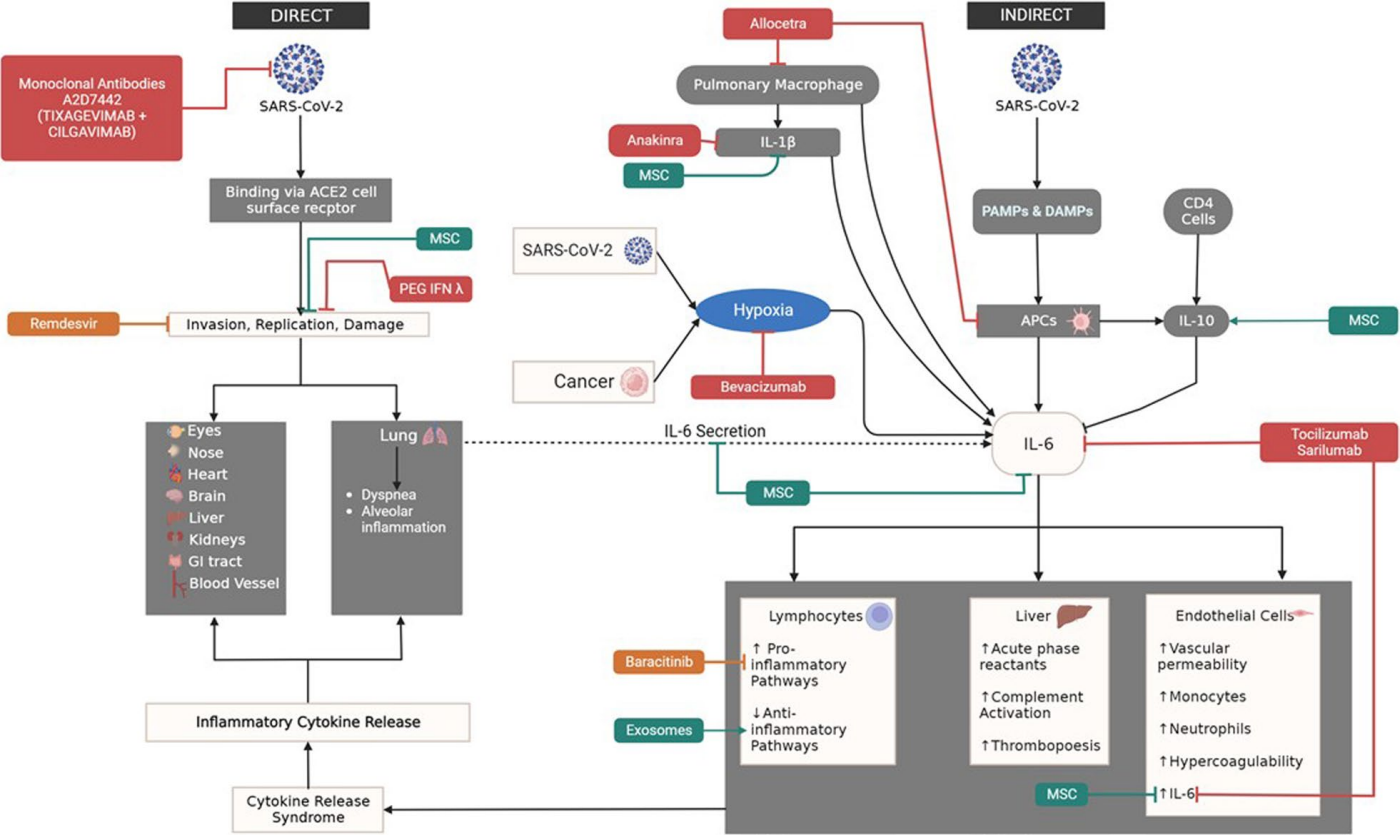
Promising therapies against severe SARS-CoV-2. Known clinical pharmacological agents are currently being tested to assess their efficacy to combat severe SARS-CoV-2. Within the context of our proposed COVID-19 Cancer Model, their mechanism of action is being highlighted. The image of SARS-CoV-2 was derived from the Centers for Disease Control and Prevention (CDC) website: https://phil.cdc.gov/Details.aspx?pid=23312

Allocetra is a universal off-the-shelf cell therapy that reprograms diseased macrophages in patients with sepsis, COVID-19, and solid tumors to their homeostatic state (Fig. 4; Table 1). It has shown promising efficacy in a Phase II investigator-initiated clinical trial against severe and critical COVID-19 in 16 hospitalized patients demonstrating an 87.5% recovery rate over an average of 5.3 days [91]. Patients were discharged post-treatment after an average of 5.6 days and exhibited a 0% mortality after 28 days. In two clinical studies in severe to critical COVID-19 patients (NCT04590053 & NCT04513470.18/21), Allocetra therapy in conjunction with standard therapy (Remdesivir, Enoxaparin, and Dexamethasone) showed early recovery with an average of 5.5 days in 21 patients [92]. In 18 patients with mild-to-severe ARDS, 16 (88.8%) demonstrated complete recovery within several days. Currently, there are no active clinical trials for the use of Allocetra in cancer patients with COVID-19.

**Table 1:** Summary of promising therapies against severe SARS-CoV-2

Therapy	Mechanism of action	Clinical trial	Clinical trial results
Convalescent Plasma	Passive immunity via neutralizing antibodies against the infectious pathogen	NCT04373460 NCT04383535 NCT04884477	Reduced the risk of disease progression leading to hospitalization in the unvaccinated Mortality benefit in immunocompromised patients with COVID-19
Allocetra	Reprogramming of diseased macrophages	NCT04590053 NCT04513470.18/21	Early recovery Reduction in hospital stay Increased survival in patients with mild-to-severe ARDS
Monoclonal antibodies (Tixagevimab–Cilgavimab)	Blocks viral attachment and entry into human cells	NCT04625725	Reduction in symptomatic COVID-19 Reduction severe and critical cases of COVID-19 Reduced mortality Minimal side effects
Peginterferon lambda	Derivative of naturally made interferons that block viral replication	NCT04354259	Reduced viral load No significant side effects
Baricitinib & Remdesivir	Baricitinib-JAK inhibitor interfering with the JAK-STAT pathway Remdesivir- nucleoside analog that inhibits the SARS-CoV-2 RNA-dependent RNA polymerase	NCT04401579 NCT04421027	Reduced recovery time Quicker improvement in clinical status especially in patients receiving ventilatory support Combination associated with fewer serious adverse events Reduction in mortality
Sarilumab/Tocilizumab	Monoclonal antibody that binds to IL-6 Receptor reducing inflammation	NCT04357860 NCT02735707	Reduction in mortality Reduction in the need for organ-support Improved clinical outcomes
Anakinra	IL-1 Receptor antagonist	NCT04318366 IRCT20120703010178N20	Reduced mortality Reduced the need for mechanical ventilation Decreased hospital stay Decreased ICU stay
Bevacizumab	Humanized vascular endothelial growth factor (VEGF) inhibitor antibody	NCT04275414	Improved blood oxygen levels within 24 h of administration Reduced duration of supportive oxygen Reduction in inflammation No deaths No severe safety concerns
Exosomal Therapy	Immune modulation and/or suppression of pro-inflammatory cytokines	NCT04384445 NCT04657406 NCT04276987	Symptomatic resolution with no disease progression by Day 30 Reduction in inflammation No adverse events
Mesenchymal Stem Cell Therapy	Stem cell therapy with immunomodulatory, anti-inflammatory, anti-fibrotic, antimicrobial, anti-viral, and fibrinolytic effects	NCT04366830 NCT04389450 NCT04614025 NCT04355728 NCT04371393	Reduction in inflammation Quicker time to extubation and hospital discharge Reduction of in-hospital mortality Few adverse events

Given that the efficacy of promising pharmacological agents are currently being tested against severe SARS-CoV-2, this table summarizes their mechanism of action, clinical trial information, and clinical trial results

### Monoclonal antibodies (Tixagevimab–Cilgavimab)

AZD7442 is a monoclonal antibody combination of Tixagevimab and Cilgavimab, which are involved in blocking viral attachment and entry into human cells (Fig. 4; Table 1). In a Phase 3 clinical trial in 5,197 adults, who either had an increased risk of exposure to SARS-CoV-2 or a decreased response to the vaccination participants, received two consecutive intramuscular injections of monoclonal antibodies—Tixagevimab followed by Cilgavimab. Results showed that symptomatic COVID-19 occurred in only 0.2% of participants compared to 1% in the placebo group [93]. The five cases of severe or critical COVID-19 and two COVID-19-related deaths all occurred in the placebo group.

A Phase 3 double-blind randomized study studying the efficacy of Tixagevimab–Cilgavimab in 1455 hospitalized patients with COVID-19 (of which 8% were immunocompromised) concluded that there was no difference between the treatment group compared to the control group (Remdesivir) in efficacy or safety [94]. However, this monoclonal antibody combination reduced hospitalization rates and no COVID-related deaths were observed in a retrospective analysis of 251 patients with B-cell malignancies when given preexposure prophylaxis [95]. The study also concluded that these patients were still at risk of breakthrough COVID-19 infections.

### Peginterferon lambda

Peginterferon lambda is a derivative of naturally made interferons within the human body as part of the innate immune response to block viral replication (Fig. 4; Table 1). Because they had demonstrated efficacy in in vitro and animal studies against SARS-CoV-2, a double-blind, placebo-controlled trial single subcutaneous injection study using peginterferon lambda was performed to assess its efficacy in 30 patients in the outpatient setting (NCT04354259) [96, 97]. By Day 7, 80% of treated patients exhibited an undetectable viral load without significant side effects [98]. The greatest benefit was seen in those patients with the high baseline viral loads.

A clinical trial using this therapy was performed in Brazil and Canada in 933 patients with COVID-19, of which a small proportion included high-risk groups (including those with cancer), concluded that the incidence of hospitalization or emergency room visits was significantly lower. However, the data were not stratified to look deeper into outcomes within the high-risk group—particularly those with active malignancy [99].

### Baricitinib & Remdesivir

Baricitinib is a JAK inhibitor that interferes with the inflammatory process. Remdesivir is a nucleoside analog that inhibits the SARS-CoV-2 RNA-dependent RNA polymerase (Fig. 4; Table 1). In a double-blind, randomized, placebo-controlled trial evaluating Baricitinib with Remdesivir in 1033 hospitalized adults with COVID-19 (NCT04401579), treatment reduced the median time to recovery from 18 to 10 days with few serious adverse effects [100]. Mortality at Day 28 after randomization was 5.1% in the treatment group compared to 7.2% in the placebo group. In a Phase 3, randomized, double-blind, placebo-controlled trial of Baricitinib plus standard of care (SOC) (remdesivir and systemic corticosteroids) compared to SOC in 101 critically ill patients with confirmed severe SARS-CoV-2 infection, showed a 39% reduction in mortality at Day 28 and 45% at Day 60 compared with 58% and 62%, respectively, in the control group (NCT04421027) [101]. However, there was no significant difference between the number of ventilator-free days or mean duration of hospitalization between the two cohorts. The rates of adverse events (measured as infections, blood clots, and cardiovascular events) were similar between both treatment groups. Despite the promising data, immunocompromised individuals (including those with cancer) were excluded.

### Tocilizumab/Sarilumab

Tocilizumab is a recombinant monoclonal antibody with a humanized murine variable domain and a human IgG1 constant domain. Tocilizumab prevents IL-6-mediated signal transduction by binding to both membrane-bound and soluble IL-6 receptors. After SARS-CoV-2 infection, immune cells (predominantly macrophages) release cytokines including IL-6, as described previously. IL-6 either attaches to its respective cell receptor (IL-6R) or the soluble receptor (sIL-6R), activating both the NF-ĸB and JAK/STAT pathways that can induce a cytokine storm. Tocilizumab binds to the IL-6R and sIL-6R resulting in a reduction of inflammatory cytokine release that can lead to a cytokine storm. Sarilumab also binds to the IL-6R but with a 15- to 22-fold higher affinity compared to Tocilizumab (Fig. 4; Table 1) [102]. Recent studies delving into the metabolomics and lipidomics of the pathophysiology of severe SARS-CoV-2 have demonstrated a downregulation of LDL-5, HDL-4, IDL, vLDL-1, and vLDL-2 and upregulation of phospholipids and Apo A2 [103]. Additionally, Anthranilic Acid was found to be upregulated in severe disease—a metabolite with poor prognostic value inversely associated with the maintenance of IL-10 and IL-18 (anti-inflammatory cytokines) [104]. This was reversed with Tocilizumab treatment.

In a randomized controlled trial comparing Sarilumab (200 mg vs 400 mg) plus SOC vs SOC alone (NCT04357860) in 115 hospitalized patients with COVID-19 showed 3 deaths in the control group, 4 deaths in Sarilumab-200 group, and no deaths in Sarilumab-400 group [105]. It was concluded that in patients with COVID-19 pneumonia and signs of systemic inflammation, a single dose of Sarilumab 400 mg was reported to have better outcomes and a strong safety profile. Despite the promising data, patients on immunosuppressive therapy and those with systemic inflammatory conditions were excluded from this study.

In a randomized controlled trial comparing Tocilizumab plus SOC with SOC (NCT04403685) in 129 hospitalized patients with severe or critical COVID-19, 17% of patients in the Tocilizumab group passed away compared with 3% in the SOC group by Day 15 [106]. The increased number of deaths prompted the trial to be stopped early. Thus, Tocilizumab plus SOC was not superior to SOC alone in improving clinical outcomes at 15 days. In an international, adaptative platform randomized clinical trial (NCT02735707), critically ill COVID-19 patients were treated with IL-6 receptor antagonists—Tocilizumab (353 patients) or Sarilumab (48 patients)—or SOC (402 patients). The IL-6 receptor antagonist group exhibited improved survival outcomes compared to control with an in-hospital mortality of 27% compared to 36% for the control group [107]. The reason for the disparities between the two trials is largely attributed to the concurrent use of steroids in the second trial, which likely helped to further dampen inflammation and reduce in-hospital mortality [108].

Although a clinical trial (NCT04370834) studying the efficacy of Tocilizumab in cancer patients with COVID-19 was terminated, a recent case report using Tocilizumab, corticosteroids, and IV immunoglobulin in immunocompromised patients with severe COVID-19 requiring mechanical ventilation demonstrated improved hemodynamic stability and were discharged without adverse events [109].

### Anakinra

Anakinra is a IL-1 receptor antagonist (Fig. 4; Table 1). In a retrospective cohort study of 392 patients (NCT04318366), 62 were treated with Anakinra, while 55 were treated with an IL-6 inhibitor. Results showed that Anakinra was associated with a significantly reduced mortality with no difference in adverse outcomes when compared to patients treated with IL-6 inhibitors [110]. In this study, steroids were used concurrently-similar to clinical studies with IL-6 inhibitors. While cancer patients were not a part of the exclusion criteria, the study did not sub-stratify outcomes within this patient population.

In a Phase 3, randomized, parallel design clinical trial in 30 patients with severe COVID-19 where 15 were treated with Anakinra or SOC for a median of 5 days (IRCT20120703010178N20), results showed that only 20% of the patients in the treatment group with Anakinra required mechanical ventilatory support in the ICU compared to 66.7% in the control group [111]. Patients treated with Anakinra also experienced a 50% reduction in hospital stay and 67% reduction in ICU stay compared to control.

A meta-analysis of four observation studies in 184 patients with early COVID-19 supported the safety of the therapy and showed that anakinra use was associated with reduction in both mortality and need for mechanical ventilation [112]. However, immunocompromised individuals (including those with malignancy) were excluded from the studies.

### Bevacizumab

Bevacizumab is a humanized vascular endothelial growth factor (VEGF) inhibitor antibody (Fig. 4; Table 1). In a single-arm clinical trial, 26 severe COVID-19 hospitalized patients were treated with either Bevacizumab plus SOC or SOC alone (NCT04275414). Results showed that patients who received Bevacizumab exhibited improved blood oxygen levels within 24 hours compared to the control group. By Day 28, 92% of the Bevacizumab treatment group did not require supportive oxygen compared to 62% for the control group. No deaths were observed in the Bevacizumab treatment group. Clinical laboratories demonstrated a reduction in inflammatory markers with no severe safety concerns [113]. Despite the promising data, immunocompromised individuals (including those with malignant tumors within 5 years of this trial) were excluded.

### Exosomal therapy

Exosomes are extracellular vesicles nanometers in size that are extracted from the culture media of cells that are able to secrete proteins and anti-inflammatory molecules (Fig. 4; Table 1). Given their anti-inflammatory potential, a proof-of-concept clinical trial was performed in 8 patients with mild-to-moderate COVID-19 who were hospitalized but not intubated (NCT04384445 & NCT04657406) and treated with Zofrin (a type of exosomal vesicle). Results showed that Zofrin treatment resulted in symptomatic resolution by Day 30 with no indication of disease progression as verified by imaging and trending inflammatory markers where both improved and decreased, respectively [114]. No adverse events were observed. However, given the small sample size and lack of placebo, these results warrant further investigation in a larger, randomized, and placebo-controlled study. Zofrin possesses efficacy against severe COVID-19 according to a recent case report whereby 3 patients with advanced disease demonstrated improved respiratory and clinical status with improved inflammatory biomarker profiles [115]. Although malignancy was not an exclusion criterion in these trials, the studies did not elaborate on outcomes within this patient population.

A similar pilot study corroborated these results. In a phase 2A single-arm, open labeled, interventional trial, seven patients with severe COVID-19 were treated with aerosolized exosomes derived from human adipose-derived MSCs for 5 days (NCT04276987). Results showed that treatment resulted in reduction in inflammation and disease progression with improvement on CT scan and no evidence of adverse events or clinical instability [116]. Given the small sample size, further studies will be needed to confirm the efficacy of this therapy in a larger cohort. Despite the promising data, individuals with a history of long-term immunosuppressive use were excluded from this study.

### Mesenchymal stem cell therapy

Mesenchymal stem cells (MSCs) are a therapeutic intervention with immunomodulatory, anti-inflammatory, anti-fibrotic, antimicrobial, and fibrinolytic effects [117]. Recent studies have also demonstrated that MSCs possess anti-viral properties through inhibition of viral entry, mRNA reverse transcription, protein translation, viral assembly, and viral exocytosis [118–120]. Given MSCs’ immunomodulatory properties where they downregulate pro-inflammatory and upregulate anti-inflammatory pathways, they are able to decrease the production of cytokines and autoantibodies and minimize the onset of hypercoagulability, especially in cancer patients [121]. This results in a downregulation of IL-6 signaling at multiple points, resulting in a net anti-inflammatory response (Fig. 4; Table 1). Early in the pandemic, MSCs demonstrated promise in Phase I clinical trials by Mesoblast (NCT04366830) and Pluristem (NCT04389450; NCT04614025), which showed an 87.5% survival rate in 8 patients with severe COVID-19 with multi-organ dysfunction after a 28-day follow-up [122, 123]. Although malignancy was not an exclusion criterion in these trials, the studies did not detail outcomes within this patient population. In a randomized, double-blinded, parallel design, placebo-controlled trial assessing the efficacy of Mesoblast’s product in 11 patients with severe COVID-19 (NCT04371393), MSC therapy resulted in quicker time to extubation (10 days) and discharge with a rapid decline in their inflammatory marker profiles [124]. Despite these promising results, patients with active malignancy (who are within 12 months of active treatment with any chemotherapy, radiation, or immunotherapy) were excluded from this study.

In a double-blind, Phase 1/2a randomized control trial (NCT04355728) in 12 patients with severe COVID-19 treated with umbilical cord-derived MSCs demonstrated a 91% overall survival and 100% survival in patients younger than 85 years old at one month after two treatments 72 hours apart [125]. The overall survival decreased to 82% at the 3-month follow-up. These results were further corroborated by two meta-analyses assessing the efficacy and safety of MSCs in treating severe/critical COVID-19 which concluded that MSCs reduced in-hospital mortality with low heterogeneity and less adverse events, improved patients’ oxygenation status, contributed to a reduction in viral load, inflammation, and mortality with improvement on radiological imaging [126, 127]. Despite the promising results, patients with active malignancy (except for those with non-melanoma skin cancer) were excluded from this study.

## Discussion

As SARS-CoV-2 becomes more endemic, targeted therapies are still lacking for severe disease. Vaccination offers a promising preventative strategy in those who respond but a long-term targeted therapy for those hospitalized is needed. Although the therapeutic options mentioned are promising, most excluded cancer patients. The few studies that included those with malignancy did not sufficiently detail outcomes within this vulnerable patient population. Because several single-agent therapies have been unsuccessful to eradicate the virus alone and may have contributed to the development of SARS-CoV-2 variants with re-infection, a strategy moving forward could utilize combination therapies that work on the direct and indirect pathways simultaneously to maximize viral control and limit systemic damage [128–133]. This is further supported by the rapid adaptive evolution of the virus to develop antigen escape through alterations in spike glycoproteins that have resulted in re-infection, enhanced replication, and transmission [129, 134, 135]. Although the CRS is likely of higher grade in cancer patients with severe SARS-CoV-2 due to greater IL-6 production, the use of combination therapies offers a promising solution to mitigate the local and systemic nature of SARS-CoV-2 and its resulting cytokine production.

Considering the endemicity of COVID-19 in multiple regions of the world, it is pivotal for healthcare professionals throughout the world to be aware of the latest developments and trends existing in the therapeutic regimen for severe disease. We hope that this study inspires the evaluation of combination therapies and expansion of previous and opening of novel clinical studies to evaluate effective therapeutics for cancer patients with COVID-19. By investing in such targeted combination interventions, cases of SARS-CoV-2 should decrease along with its associated long-term sequelae and thereby allow cancer patients to resume their treatments with minimal interruption [136].

## Conclusion

SARS-CoV-2 is a highly transmissible betacoronavirus whose rapid spread has caused a global pandemic and drastically altered daily life. Cancer patients due to their immunocompromised status are especially susceptible. We presented a model system for direct and systemic damage for SARS-CoV-2 and its interplay with malignancy. This synergism will result in higher grade CRS that will require combination anti-inflammatory therapies. By adopting treatment strategies from anticancer therapies and HIV antiretroviral therapy, systemic combination therapies offer the most promising means of limiting systemic inflammation and viral progression with protection against treatment resistance.
